# The Intriguing World of Vascular Remodeling, Angiogenesis, and Arteriogenesis

**DOI:** 10.3390/ijms25126376

**Published:** 2024-06-09

**Authors:** Paul H. A. Quax, Elisabeth Deindl

**Affiliations:** 1 Einthoven Laboratory for Experimental Vascular Medicine, Department of Surgery, Leiden University Medical Center, 2300 RC Leiden, The Netherlands; 2 Walter-Brendel-Centre of Experimental Medicine, University Hospital, Ludwig-Maximilians-Universität, 81377 Munich, Germany; 3 Biomedical Center, Institute of Cardiovascular Physiology and Pathophysiology, Ludwig-Maximilians-Universität München, Planegg-Martinsried, 82152 Munich, Germany

Vascular remodeling is a very general feature related to angiogenesis and arteriogenesis, which are involved in neovascularization processes. Vascular remodeling is of course a crucial element in development [1,2], but also in pathological vascular remodeling processes such as atherosclerosis [3] and aneurysm formation.

In this Special Issue, many aspects of vascular remodeling are addressed. Zebrafish are an excellent model for studying blood vessel development [4,5]. The role of small GTPases in vascular development in zebrafish is addressed by Urade et al. [6], who describe the role of the small GTPase subfamilies in the development of the vascular system in zebrafish. Critical processes in blood vessel development in zebrafish relate to vascular endothelial growth factor (VEGF) signaling, Notch signaling, and bone morphogenetic protein (BMP) signaling [7]. Impaired small GTPases can contribute to vascular remodeling [8]. Despite this importance, their regulatory role in vascular development is unclear. This review discusses the role of the small GTPases along with their regulators in blood vessel development in zebrafish [6].

Another group of small regulators of vascular remodeling are microRNAs. microRNAs are regulators for multifactorial processes such as angiogenesis, arteriogenesis, and vascular remodeling [9,10]. Evidence on the regulation of vascular remodeling by various microRNAs is still growing. Here, Wang et al. [11] studied the role of microRNA-30b in the regulation angiogenesis. They show that miR-30b appears both necessary and sufficient for IL21/IL-21R-mediated angiogenesis and may present a new therapeutic option to treat peripheral arterial disease [12] if IL21R is not available for activation.

The stimulation of angiogenesis and neovascularization by different cells with a pro-angiogenic character remains very attractive from a therapeutic point of view [13,14]. Gatina et al. focused on umbilical-cord-derived mononuclear cells and genetically modified them using adenoviral constructs, adVEGF, adFGF2, and SDF1, and utilized adGFP as a control. They showed that these genetically modified UBC-MC were able to induce neovascularization in in vivo Matrigel plug tubule formations in mice [15].

Shear stress can generally be recognized as a crucial trigger in vascular remodeling processes [16]. In this Special Issue, Motlana et al. [17] describe how a computational fluid dynamics model can be used for analyzing the role of shear stress in angiogenesis in rheumatoid arthritis, as it represents an important factor [18]; they show that the magnitude of wall shear stress relates to the degree and extent of new blood vessel development.

Next to angiogenesis, arteriogenesis plays a crucial role in neovascularization [19,20]. However, the processes of angiogenesis and arteriogenesis, although different at regulatory levels [21,22], cannot be regarded as non-related processes. Le Noble and Kupatt show, very elegantly and convincingly, that these two processes, arteriogenesis and angiogenesis, are two interdependent processes in development and disease [23]. They describe the mechanisms and signals that contribute to the synchronized growth of micro- and macrovascular structures after ischemic challenges, as well as during development. They conclude that a long-term successful revascularization strategy should aim to both remove obstructions in the proximal part of the arterial tree and restore “bottom-up” vascular communication based on striking similarities between micro- and macrovascular structures.

The induction of arteriogenesis is a process that attracts a lot of attention from the clinical point of view in relation to peripheral arterial disease (PAD) patients [14,24], as good therapeutic options are still lacking. The link with the immune system [25,26,27] and the innate immune system, in particular [27,28,29,30], has raised a lot of attention with regard to new therapeutic options. In this Special Issue, Götz et al. provide some compelling data how cobra venom factor, known to be a very potent inhibitor of Complement Factor C3 [31], is able to boost arteriogenesis in mice after femoral artery ligation, demonstrating the role of the innate immune system in arteriogenesis [32]. Furthermore, the importance of the adaptive immune system in arteriogenesis is shown in the study by Kumaraswami et al. [33], who found an impaired arteriogenic response in mice deficient in Rag1, i.e., lacking both T cells and B cells. This is in line with previous work supporting the role of T and B cells [25,34,35]. In this Special Issue, Wong et al. study the role of tripartite-motif-containing protein 2 (TRIM2) in vascular remodeling and did not observe any effects on blood flow recovery in a hind limb ischemia model of TRIM2-deficient mice, despite the fact that TRIM2 knockdown in endothelial cells in vitro attenuated the inflammation-driven induction of critical angiogenic mediators.

Despite cell therapy being regarded as a promising approach for inducing neovascularization in PAD patients, many clinical trials using autologous bone marrow transplantation to induce neovascularization have failed or yielded ambiguous results [36,37]. In an attempt to unravel the effect of human-bone-marrow-derived mononuclear cells on angiogenesis, Peeters et al. studied the effect of human mononuclear cells on angiogenic responses of human cells in a large set of in vitro angiogenesis models. Despite their extensive studies, in which proliferation, migration, tube formation and aortic ring assays were studied, none of the conditions tested showed a pro-angiogenic effect of human-bone-marrow-derived cells [38].

Another very important aspect of vascular remodeling is the formation and maturation of arteriovenous fistulas (AVFs), required for vascular access in dialysis patients. It is well known that these AVFs fail very frequently [39,40]. In this Special Issue, Laboyrie et al. review the role of the extracellular matrix in AVF maturation. Moreover, they examine the effect of chronic kidney failure on vasculature remodeling and ECM changes post AVF surgery, and describe current ECM interventions to improve AVF maturation. Furthermore, they discuss the suitability of ECM interventions as a therapeutic target for AVF maturation [41].

Lastly, this Special Issue contains a paper by Anis et al. [42] that describes the vascular remodeling processes in pulmonary hypertension; more specifically, how non-muscle MLCK contributes to endothelial cell hyperproliferation in vascular remodeling in these pathological processes.

## Conclusions

In conclusion, in this Special Issue we hope to have demonstrated the broad role of vascular remodeling in pathological and physiological processes, and we have illustrated some of the complex regulatory mechanisms involved. Moreover, we discuss several potential therapeutic targets for intervening in vascular remodeling.
